# OCT Assisted Quantification of Vitreous Inflammation in Uveitis

**DOI:** 10.1167/tvst.11.1.3

**Published:** 2022-01-04

**Authors:** Xiaoxuan Liu, Aditya U. Kale, Giovanni Ometto, Giovanni Montesano, Alice J. Sitch, Nicholas Capewell, Charlotte Radovanovic, Nicholas Bucknall, Nicholas A. V. Beare, David J. Moore, Pearse A. Keane, David P. Crabb, Alastair K. Denniston

**Affiliations:** 1 Academic Unit of Ophthalmology, Institute of Inflammation and Ageing, College of Medical and Dental Sciences, University of Birmingham, UK; 2 Department of Ophthalmology, University Hospitals Birmingham NHS Foundation Trust, Birmingham, UK; 3 Moorfields Eye Hospital NHS Foundation Trust, London, UK; 4 Health Data Research UK, London, UK; 5 Birmingham Health Partners Centre for Regulatory Science and Innovation, University of Birmingham, Birmingham, UK; 6 Optometry and Visual Sciences, City, University of London, London, United Kingdom; 7 NIHR Biomedical Research Centre, Moorfields Eye Hospital NHS Foundation Trust and UCL Institute of Ophthalmology, London, UK; 8 NIHR Birmingham Biomedical Research Centre, University Hospitals Birmingham NHS Foundation Trust and University of Birmingham, UK; 9 Institute of Applied Health Research, University of Birmingham, UK; 10 Patient Involvement Group in Uveitis, Birmingham, UK; 11 Department of Eye and Vision Science, University of Liverpool, UK; 12 Institute of Ophthalmology, University College London, London, UK

**Keywords:** vitreous haze, imaging, optical coherence tomography, uveitis, inflammation

## Abstract

**Purpose:**

Vitreous haze (VH) is a key marker of inflammation in uveitis but limited by its subjectivity. Optical coherence tomography (OCT) has potential as an objective, noninvasive method for quantifying VH. We test the hypotheses that OCT can reliably quantify VH and the measurement is associated with slit-lamp based grading of VH.

**Methods:**

In this prospective study, participants underwent three repeated OCT macular scans to evaluate the within-eye reliability of the OCT vitreous intensity (VI). Association between OCT VI and clinical findings (including VH grade, phakic status, visual acuity [VA], anterior chamber cells, and macular thickness) were assessed.

**Results:**

One hundred nineteen participants were included (41 healthy participants, 32 patients with uveitis without VH, and 46 patients with uveitis with VH). Within-eye test reliability of OCT VI was high in healthy eyes and in all grades of VH (intraclass correlation coefficient [ICC] > 0.79). Average OCT VI was significantly different between healthy eyes and uveitic eyes without and uveitic eyes with VH, and was associated with increasing clinical VH grade (*P* < 0.05). OCT VI was significantly associated with VA, whereas clinical VH grading was not. Cataract was also associated with higher OCT VI (*P* = 0.03).

**Conclusions:**

OCT VI is a fast, noninvasive, objective, and automated method for measuring vitreous inflammation. It is associated with clinician grading of vitreous inflammation and VA, however, it can be affected by media opacities.

**Translational Relevance:**

OCT imaging for quantifying vitreous inflammation shows high within-eye repeatability and is associated with clinical grading of vitreous haze. OCT measurements are also associated with visual acuity but may be affected by structures anterior to the acquisition window, such as lens opacity and other anterior segment changes.

## Introduction

Uveitis is a significant cause of visual impairment, accounting for approximately 10% to 15% of total blindness in the developed world.^1^^,^^2^ The term describes a broad range of syndromes characterized by intra-ocular inflammation, which can be divided anatomically into anterior, intermediate, posterior, and panuveitis. Assessment of uveitic inflammatory activity is challenging, owing to the heterogeneity of phenotypes and underlying etiology, as well as a lack of sensitive and reliable measures of disease.^3^ Even today, measuring uveitic activity is through subjective clinician assessment using grading systems based on ordinal scales.^4^ For vitreous haze, a key marker of inflammation and a common outcome measure of choice for effectiveness trials, this is done using the National Eye institute vitreous haze (NEI VH) scale, a six point scoring system for grading inflammatory activity.

Recent technological advancements, in particular, optical coherence tomography (OCT), have enabled a more objective, test-based approach to retinal disease detection and monitoring. We first described the use of OCT-based analysis of vitreous inflammation in 2014.^5^ The earlier technique involves calculation of mean pixel intensity for the vitreous space versus the retinal pigment epithelium (RPE) to yield a ratio, the Vit-RPE relative intensity index. A significant difference in the Vit-RPE relative intensity index was detectable in eyes with, and those without, vitreous haze. In 2016, Sreekantam et al. reported the ability of the Vit-RPE relative intensity index to detect treatment response in a retrospective analysis of patients with uveitic cystoid macular oedema receiving sub-Tenon's triamcinolone acetonide (STTA).^6^ This retrospective analysis of 22 patients demonstrated the ability of the Vit-RPE relative intensity index to detect a significant treatment response in uveitic eyes with cystoid macular edema. Since then, we have made further refinements to our technique by defining optimum acquisition settings to minimize variation in measurements due to noise.^7^ Given the reliance on RPE relative intensity, a recognized limitation of this technique was confounding of the intensity signal in cases of RPE disruption (such as in the case macular oedema and choroidal neovascular membrane). We have therefore moved away from reliance on the Vit-RPE ratio and instead adopted a new approach based on quantifying the total raw OCT signal in the vitreous space.

This study is the first prospective evaluation of OCT-measured vitreous intensity (VI) to date. First, the study aims to estimate the reliability of the OCT measurements within healthy eyes and uveitic eyes. Second, the study aims to determine the clinical validity of the OCT-based technique by assessing its association with clinical measurements of inflammation and visual function in uveitic eyes.

## Methods

### Participant Recruitment

Participants were prospectively recruited from January 2018 to December 2019 at University Hospitals Birmingham NHS Foundation Trust, Birmingham, UK. Healthy controls were hospital staff or family members/carers of patients with no history of eye disease attending the ophthalmology outpatient department. Healthy controls were assessed by undilated slit-lamp examination to rule out any existing ophthalmic disease. Patients attending the uveitis clinic, with a diagnosis of posterior-segment involving uveitis were consecutively enrolled into the study as uveitic eyes. All patients with a diagnosis of posterior segment involving uveitis were included. Exclusion criteria included those younger than 16 years and those where the diagnosis was subsequently confirmed to not be uveitis (including masquerade syndromes, such as lymphoma). Informed consent was given by all participants. This protocol was approved by the London-South East Research Ethics Committee (18/LO/1332). This protocol adhered to the tenets of the Declaration of Helsinki.

### Image Acquisition

**Figure 1.**
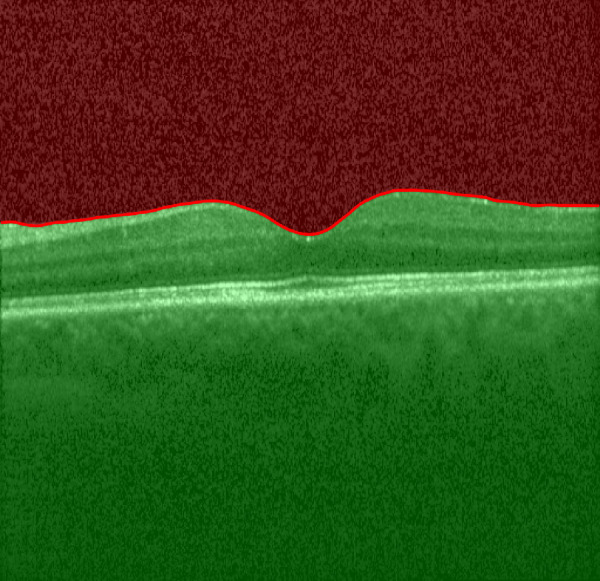
Segmented B scan with region of interest, vitreous. A ratio of the signal intensity between the red area and the whole B scan (red plus green areas) yields a measurement of vitreous signal intensity which is logarithmically transformed and recorded in arbitrary units.

OCT scans were performed using the Heidelberg SPECTRALIS OCT (Heidelberg Engineering, Heidelberg, Germany) using a 30-degree lens, as per a prespecified protocol (7 horizontal 20-degree B scans at ART 9, centered on the macula, covering a theoretical area of 20 degrees by 5 degrees [5.9 mm by 1.5 mm] and with the macula positioned in the middle of the B scan). For each participant, the eye was scanned three consecutive times by an experienced OCT technician prior to clinical examination. The pupils were pharmacologically dilated for patients with uveitis as part of standard clinical practice. Healthy participants did not receive dilating drops, as OCT scans can typically be acquired in undilated healthy eyes. The OCT technician did not have prior knowledge of the patient's disease history or clinical status. Each OCT volume was exported in the .vol format at the end of the study. The OCT VI is automatically derived for each B scan through our custom algorithm implemented in Matlab (The MathWorks, Natick, MA, USA). First, the vitreous space is automatically segmented by our segmentation tool ReLayer.^8^ Then the raw signal of each OCT B scan is obtained from .vol files exported from the Heidelberg Eye Explorer. The VI measurement is calculated as the log transform of the ratio of the signal in the vitreous (Fig. 1, in red) to the signal from the whole B scan (see Fig. 1, red and green areas).

### Clinical Examination

At each routine clinical visit, patients with uveitis had visual acuity tested (Snellen chart at 6 meters) and underwent routine dilated ophthalmic examination by an experienced uveitis specialist (author A.K.D.). The disease locality, anterior chamber (AC) cell grade, as per the Standardization of Uveitis Nomenclature (SUN) grading system,^4^ phakic status (phakic, pseudophakic, or cataract), vitreous haze grade (NEI VH grading, assessed through indirect ophthalmoscopy) were recorded. Additionally, the OCT central subfield thickness (CST), as automatically calculated by Heidelberg Eye Explorer, was taken as a measure of macular thickness. All OCT analysis for VI was carried out at the end of the study, therefore, the uveitis specialist had no knowledge of the OCT-derived VI measurement.

### Statistical Analysis

#### Test Reliability

A multilevel mixed model was used for this analysis to account for the clustering (mean of several B scans per volume and three repeated volumes per subject). The intraclass correlation (ICC) at the within subject-eye and between subject-eye levels were calculated for healthy eyes and uveitic eyes separately. For uveitic eyes, the ICC across the whole cohort, as well as separated by NEI VH grade, is reported. ICC is interpreted across a range based on 95% confidence interval (CI).^9^

#### Ability to Discriminate Eyes With and Without Inflammation

The study cohort was split into three groups: healthy subjects, uveitic eyes with no VH (NEI VH grade 0), and uveitic eyes with VH (NEI VH grades 0.5 and above). The VI measure for seven B scans in each volume was averaged to derive a single measurement per volume, and the mean of the three repeated volumes was used as the final measure for each eye. A simple linear regression was fitted, with the OCT vitreous haze measurement modeled as the outcome and the three groups treated as a categorical independent variable.

#### Association With NEI VH Grading

To assess the association between the OCT VI measurement with NEI VH grading, the mean difference of the OCT vitreous measurement at each NEI VH grade was calculated compared to the reference measurements from the healthy control group. The NEI VH grading was treated as a categorical independent variable and OCT vitreous measurement modeled as the outcome. The Wald Test was used to assess for overall significance of the difference between OCT intensity measurements in each NEI VH grade.

#### The Effect of Age, Phakic Status, and AC Cells on the OCT Vitreous Intensity Measurement

The effects of age, phakic status (grouped as cataract present or absent), and AC cells (SUN grade 0 versus SUN grade 0.5+ and above) on the OCT VI measurement was evaluated while adjusting for severity of inflammation, as per the NEI VH grade (categorized as NEI VH grade 0, 0.5+, 1+, or 2+ and above). The OCT VI measurement was modeled as the outcome and predictors were age, phakic status, AC cells, and NEI VH grading.

#### Ability of OCT Vitreous Intensity to Predict VA and CMT

To explore whether the OCT VI measurement can predict VA and CMT, two separate multivariate analyses were conducted. In both analyses, OCT VI and NEI VH grading are treated as predictors, with NEI VH grading grouped as grade 0, 0.5+, 1+, and 2+ and above. In the first multivariate analysis, which models VA as the outcome, age, phakic status (grouped as cataract present or absent), and CMT are included as predictors. VA was converted from Snellen to logMAR (counting fingers [CFs] and hand movements [HMs] were converted to 2.1 and 2.4 logMAR, respectively).^10^ In the second multivariate analysis where CMT is modeled as the outcome, AC cell grading (grouped as AC cells present/absent) and NEI VH grading are treated as predictors, with NEI VH grading grouped as grade 0, 0.5+, 1+, and 2+ and above.

One eye per participant was included in the analysis. As patients with active vitreous inflammation were uncommon, for all analyses, the eye with inflammatory activity (defined as NEI VH grade 0.5+ or presence of cystoid macular oedema, retinal vasculitis, or chorioretinal inflammation) was purposefully selected to oversample this cohort. In the absence of disease activity, or if both eyes showed active disease, the right eye was selected. All statistical analysis was performed in Stata 16 (StataCorp LLC, College Station, TX, USA).

## Results

### Participant Characteristics

**Figure 2.**
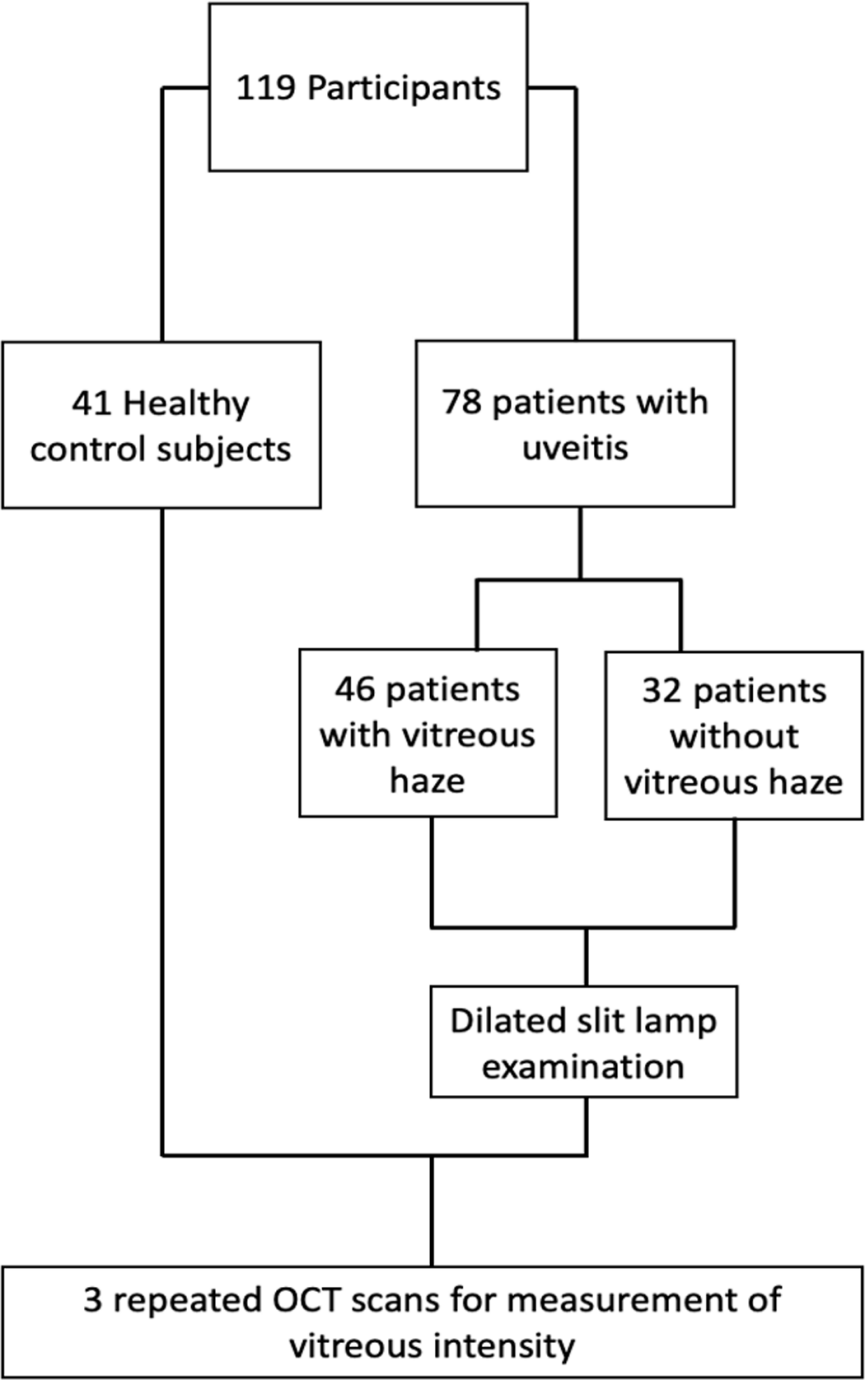
Flowchart showing patient recruitment and assessment process.

In total, 119 people participated in this study, including 41 healthy control subjects, 46 subjects with VH in one or both eyes and 32 without VH in either eye. The division of participants into groups is shown in Figure 2. One participant with grade 4+ VH (NEI VH scale) was excluded from the analysis as the vitreous density was so severe that an OCT image could not be acquired. Participant characteristics are summarized in Table 1. Mean VI (SD) for test participants without haze was –24.2 (SD = 1.5) and for participants with haze was –22.8 (SD = 1.9). Control participants included 16 (39%) men and 25 (61%) women with a mean age of 48 years (SD = 19). The mean (SD) VI value across all healthy eyes was –25.04 (SD = 1.77).

**Table 1.** OCTAVE Study: Characteristics of Recruited Patients With Uveitis

	Uveitis Without	Uveitis With
	Haze (32)	Haze (45)
**Sex**		
Male	11 (34%)	17 (38%)
Female	21 (66%)	28 (62%)
**Mean age (SD) in years**	52 (+17)	51 (+18)
**Ethnicity**		
White	21 (66%)	33 (73%)
Asian/Asian British	6 (19%)	10 (22%)
Black/African/Caribbean/Black British	2 (6%)	2 (4%)
Mixed/Multiple ethnicgroups	3 (9%)	0 (0%)
**Etiology**		
Idiopathic	16 (50%)	22 (49%)
Behçet's disease	1 (3%)	0 (0%)
Birdshot chorioretinitis	0 (0%)	3 (7%)
Cytomegalovirus retinitis	0 (0%)	1 (2%)
Fuchs’ heterochromiccyclitis	0 (0%)	1 (2%)
Herpes zosterophthalmicus	2 (6%)	2 (4%)
HLA-B27 associateduveitis	2 (6%)	2 (4%)
Juvenile idiopathicarthritis	1 (3%)	3 (7%)
Multiple sclerosis	1 (3%)	1 (2%)
Tuberculosis	1 (3%)	1 (2%)
Multifocal choroiditis	1 (3%)	0 (0%)
Punctate innerchoroidopathy	1 (3%)	0 (0%)
Retinal vasculitis	2 (6%)	0 (0%)
Sarcoidosis	3 (9%)	8 (18%)
Toxoplasmosis	1 (3%)	1 (2%)
**Phakic status**		
No cataract	12 (38%)	17 (38%)
Cataract	11 (34%)	15 (33%)
Pseudophakic	9 (28%)	13 (29%)
**Anatomic** **subtype of uveitis**		
Anterior	3 (9%)	5 (11%)
Intermediate	15 (47%)	16 (36%)
Posterior	4 (13%)	7 (16%)
Panuveitis	10 (31%)	17 (38%)

### Within and Between Eye Measurement Variability

The within-eye ICC was 0.79 (95% CI = 0.71–0.85) and the between-eye ICC was 0.75 (95% CI =0.65–0.86) in healthy participants. In uveitic eyes, the between-eye ICC was > 0.79 in all grades of VH in the uveitic eyes (however, CI ranges were from 0.46 to 0.99). Table 2 shows the within-participant eye reliability separated by NEI VH grade.

**Table 2.** Within-Participant Eye ICC from the Three Repeated Measurements in Eyes With Vitreous Haze of Varying Severity (as Assessed Using the NEI VH Scale)

NEI Vitreous	Number Of	Within-Participant
Haze Grade	Eyes	Eye ICC (95% CI)
0	32	0.83 (0.75–0.89)
0.5+	25	0.90 (0.83–0.94)
1+	13	0.89 (0.78–0.95)
2+	4	0.79 (0.46–0.94)
3+	3	0.93 (0.72–0.99)

### Ability to Detect Different Levels of Vitreous Haze

**Figure 3.**
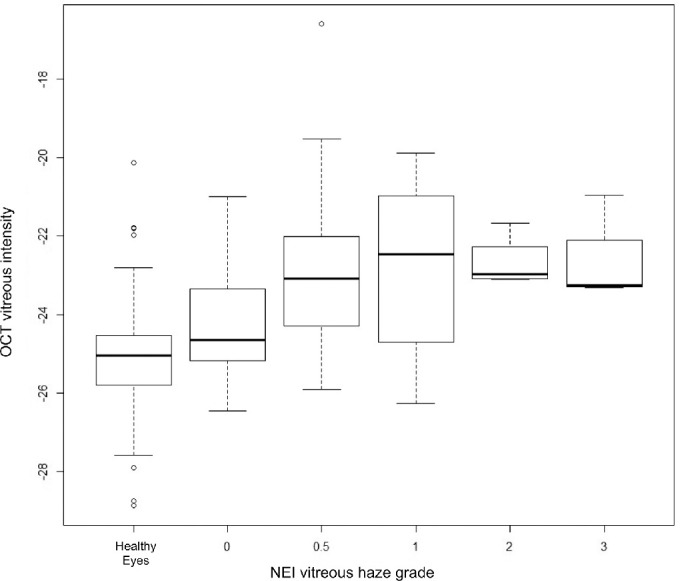
Box and whisker plot showing median of OCT vitreous intensity measurements in each NEI vitreous haze grade. Minimum point is 1.5 × IQR below first quartile, and maximum point is 1.5 × IQR above first quartile. *Circles* denote outliers.

Both uveitic eyes with and without haze showed significantly different OCT vitreous intensities compared with healthy eyes (Table 3). In the uveitic eyes, the OCT VH measurement was significantly increased at NEI VH grades 0, 0.5, 1, 2, and 3 compared with measurements obtained from healthy controls (*P* < 0.005; Supplementary Table S1). Median VI (interquartile range [IQR]) for participants with grade 0 was –24.6 (+1.8), for grade 0.5+ was –23.1 (+2.5), for grade 1+ was –22.5 (+3.8), for grade 2+ was –23.0 (+1.1), and for grade 3+ was –23.3 (+2.4; Supplementary Table S2; Fig. 3).

**Table 3.** Mean OCT Vitreous Intensity Score in Eyes With and Without Vitreous Haze.

	Number	Mean OCT
Group	Of Eyes	VI (SD)
Healthy eyes	41	–25.0 (+1.77)
Uveitic eyes - quiescent (NEI vitreous haze grade 0)	32	–24.2 (+1.46)^*^
Uveitic eyes - active (NEI vitreous haze grade 0.5 and above)	45	–22.8 (+1.87)^**^

* *P* < 0.05.

** *P* < 0.005 in the simple linear regression.

### Effects of Age, Phakic Status, and AC Cells on the OCT Vitreous Intensity Measurement

**Figure 4.**
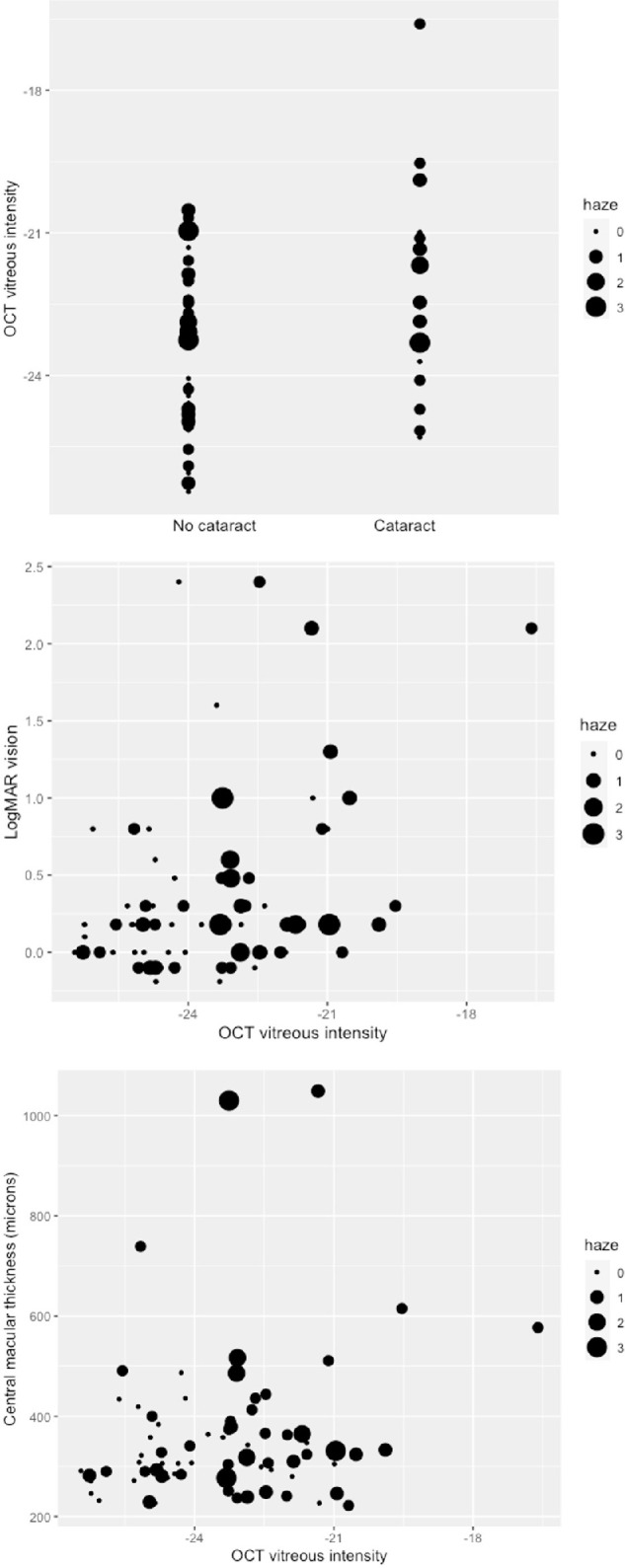
OCT vitreous intensity in eyes with and without cataract (*top*), association between OCT vitreous intensity measurement with visual acuity (LogMAR) (*middle*), association between OCT vitreous intensity measurement with central macular thickness (*bottom*).

In the multivariate analysis, the presence of cataract was shown to have a significant association with increased OCT vitreous measurement (*P* = 0.03) when adjusting for NEI VH grade (Supplementary Table S3; Fig. 4). No significant association was detected between the OCT vitreous measurement and patient age (*P =* 0.05) or presence/absence of AC cells (*P =* 0.88).

### Ability to Predict VA and CMT

In the multivariate analysis where VA was modeled as the outcome, the OCT VI measure showed a modest, albeit significant association with VA (*P* = 0.04, 95% CI = 0.003–0.145; see Fig. 4), whereas no association could be found with the NEI VH (*P* = 0.17, *P* = 0.70, and *P =* 0.07, respectively, for grades 0.5+, 1+, and 2+ and above). A strongly significant association was found between CMT and VA (*P* < 0.005). No association was found between phakic status and VA (*P* = 0.65). See Supplementary Table S4 for the mean visual acuities by NEI VH grades.

In the multivariate analysis where CMT was modeled as the outcome, neither the OCT VI measure (*P* = 0.35; see Fig. 4) or the NEI VH grades (*P* = 0.33 for grade 0.5+, *P* = 0.99 for grade 1+, and *P* = 0.04 for grade 2+ and above) were associated with CMT. Presence of AC cells also did not show association with CMT (*P* = 0.14).

## Discussion

This study is the first prospective clinical evaluation of the OCT VI measurement in uveitic and healthy eyes. We observed moderate to high reliability of this test throughout the study in both the eyes of healthy participants and patients with uveitis.^9^^,^^11^ Specifically, with the study protocol of a single operator taking repeated images on the same visit, within-eye ICC was 0.79 and between-eye reliability was 0.75. In patients with uveitic eyes, we found within subject test-retest variability of the measurement to be comparable with healthy eyes (ICC > 0.79 in all grades of NEI VH) and that the mean measurement was significantly different between groups of healthy eyes and uveitic eyes without VH and uveitic eyes with VH. OCT VI also showed an association with NEI VH grading, with measurements in each increasing NEI VH grade showing significant incremental increase in OCT intensity. However, substantial overlaps in the individual OCT measurement were observed between NEI VH grades. These overlaps may be attributable to poor signal-to-noise ratio from the OCT technique, but could also be attributable to the imprecision and poor reliability of clinician-based grading using the NEI VH scale. Potential causes of measurement variability (noise), which can disrupt the OCT measurement include non-inflammatory features, such as tear film irregularities, inadequate pupillary dilation, and cataracts; Or inflammatory causes, such as keratic precipitates, AC cells and flare, posterior synechiae, and vitreous cells. On the other hand, poor reliability of the clinical grading system, due to its subjectivity, can make it unreliable and also explain why misclassifications can occur.

Presence of cataract or other light scattering media opacities can increase the OCT vitreous measurement given the underlying principle of the OCT technique, which measures this scattering effect caused by vitreous debris and exudates. Aging and AC cells have the potential to increase light scatter, however, there was no association found in this study.^12^ In the multivariate analysis, we were able to detect a small but statistically significant association between the OCT vitreous measurement and visual acuity, whereas none could be shown for NEI vitreous grading and VA. This association was significant even after adjusting for other potential confounders of reduced VA, including phakic status and CMT. This suggests the OCT vitreous measurement may be a better predictor of visual function than the NEI VH clinical grading system and is in keeping with the findings of Sreekantam et al., who reported a significant correlation between VA and OCT Vit-RPE relative intensity ratio.^6^

The OCT vitreous measurement did not demonstrate an association with CMT. This could be explained in several ways. First, there may be no consistent biological association between the presence and severity of VH density and macular edema. Second, whereas CMT and vitreous inflammation can co-exist, macular edema may develop following some time after VH (or vice versa) and therefore the association cannot be detected cross-sectionally. Third, the resolution of VH may occur faster than CMT (or vice versa) and the window of opportunity to detect both signs at their peak manifestation is relatively small. To understand the time course relationship between VH and macular edema (or indeed any other manifestation of inflammation), a longitudinal study with close time points during active flares is needed.

The main strength of this study is that it is the first prospective evaluation of the OCT vitreous measurement in a real-world setting. In comparison to previous retrospective studies, a number of potentially confounding factors could be controlled for, such as ensuring the same uveitis specialist was performing the ophthalmic examination in a standardized way and assessing all VH grades as per the NEI VH scale. Additionally, the scan protocol was predefined and based on prior evidence of the most reliable technique, instead of previous retrospective studies, which accepted routine care OCT scan protocols. The association between Vit-RPE VI and CMT and VA have previously been explored by Sreekantam et al.,^6^ however, this study was also the first to consider its predictive properties in the context of other potential confounders, such as age, phakic status, and AC cells.

Several limitations of this study should be noted. First, the number of eyes with significant vitreous inflammatory activity was small. As the study setting was a routine follow-up uveitic clinic, where the majority of patients were being monitored for stable disease, active uveitic inflammation was uncommon. Despite attempts to increase the proportion of eyes with higher levels of VH by purposely sampling the inflamed eye in each patient, the majority of included eyes had VH grades 0 to 1+ and only 7 out of 77 eyes had grade 2+ and above. Second, only one experienced uveitis specialist performed the clinical assessment, including assessing the NEI VH grade. This was because only one uveitis specialist experienced in VH grading was available for the entire duration of the study. Third, several relevant markers of inflammation, such as AC flare, were not recorded or quantified in this study. These were not included in the study protocol but would have provided a more complete clinical picture of the inflammatory disease state. Fourth, although a wide range of uveitic entities (including those which typically exhibit retinal anatomic changes) were included in this study, no formal evaluation on the effect of retinal changes on the VI signal was performed. As the image analysis includes quantification of the reflectivity anterior and posterior to the internal limiting membrane, it is possible that retinal structures with varying reflectivity may confound the signal.

There are several possible explanations for the wide variability seen within each NEI VH grade. It may be attributable to the poor reliability of the clinical grading system itself, but there are also technical factors which may limit the clinical validity of our current approach. First, only a small part of the vitreous is sampled by the current scanning method. The theoretical area sampled is 5.9 mm by 1.5 mm, which represents only a small volume of the entire vitreous (estimated total volume 4.6–4.9 mm3)^13^ and most of the anterior vitreous is unaccounted for. The distribution of VH along the axial length of the eye is not well understood, other than in certain uveitic entities (such as toxoplasmosis), which present with patches of VH directly overlying areas of focal chorioretinitis.^14^ Focal patches of VH could be missed by our current approach, which focuses at the macula, in order to avoid distortion caused by refractive properties in noncentral areas and achieve the most focused image.

Second, in the same way that anterior vitreous is not captured by the current method, neither are other anterior segment ocular structures, which may disrupt the OCT signal and confound the measurement. For example, media opacity, such as cataracts, are known to have a light scattering effect on OCT and causes degradation of the image quality.^15^^,^^16^ Similar effects are likely caused by other anterior segment opacifications along the scan axis, such as keratic precipitates, AC cells, AC flare, posterior synechiae, vitreous cells, and vitreous syneresis. The current measurement acquired from the current technique assumes that all signal degradation is caused by vitreous opacity and cannot adjust for the impact of anterior structures. Future work by this group will aim to explore how larger vitreous areas can be captured and how reflectivity caused by anterior structures can be accounted for.

Further work is required to ascertain the measurement variability over time, which will be important for determining diagnostic thresholds for healthy and disease states. Additional evidence is also required for determining the sensitivity of OCT VI for detecting the treatment effect. One potential approach to do this is to nest a silent test evaluation study within a therapeutic trial. For example, most clinical trials in uveitis already collect the necessary outcomes at each visit: OCT macula scans, clinician-based measures of inflammatory activity (including NEI VH scale, AC cells/flare, presence/absence of new inflammatory chorioretinal lesions, and other markers of improving or worsening disease). The addition of an observational analysis, which would support the clinical validation of a new test or biomarker, would be relatively easy to do without negatively impacting upon the trial conduct.

In conclusion, this study builds upon our previous work and provides further evidence supporting the validity of an OCT-derived marker of vitreous inflammation. There is an urgent need for more accurate and sensitive markers of inflammation, not only to support better clinical judgment and decision making in clinical care, but also to detect treatment effects in therapeutic trials and bring new potentially effective therapies to patients. OCT has not only shown potential for quantifying vitreous inflammation in our work, it can also be used to measure other key inflammatory components including AC cells, AC flare (both anterior segment OCT), macular thickness, and focal inflammatory lesions.^17^^–^^19^ Additionally, OCT acquisition is noninvasive, fast, and simple, and can be provided in community settings such as high street optometrists. In many ways, OCT has the potential to provide an all-in-one comprehensive examination of intraocular inflammation, providing clinicians and patients with truly objective and quantifiable metrics, ideal for longitudinal disease monitoring.

## Supplementary Material

Supplement 1
