# Supplementary material for: OCT Assisted Quantification of Vitreous Inflammation in Uveitis
Source: Transl Vis Sci Technol. 2022 Jan 4;11(1):3. doi: 10.1167/tvst.11.1.3 (PMC8742534; doi:10.1167/tvst.11.1.3)
Supplement: Supplement 1 [file tvst-11-1-3_s001.pdf]

Supplementary Materials

Supplementary table 1. Mean OCT Vitreous Intensity at different AC cell Grades

AC Cell Grade	Number of Eyes	Mean OCT vitreous intensity (SD)
0	49	-23.6 (1.65)
0.5	17	-23.1 (2.26)
1	2	-24.0 (1.09)
2	4	-23.9 (1.62)
3	3	-20.7 (0.75)
4	1	-21.0 (-)

*Supplementary table 2. Mean difference of OCT vitreous intensity at NEI vitreous haze grades 0.5+ to 3+ compared to grade 0. (*reference grade 0)*

p<0.05, **p<0.005 in the Wald Test

NEI vitreous haze*	Number of Eyes	Mean difference above reference (95% CI)
Healthy eyes	41	Reference
0	32	1.01 (0.42, 1.61)*
0.5	25	2.47 (1.83, 3.11)**
1	13	2.61 (1.81, 3.42)**
2	4	2.52 (1.20, 3.85)**
3	3	2.28 (0.76 - 3.79)*

Supplementary table 3. Effect of phakic status on OCT vitreous intensity (Only one eye for grades 2 and 3 where cataract was present).

NEI vitreous haze grade	Mean OCT vitreous intensity (SD)	
	Cataract not present	Cataract present
0	-24.4 (1.44)	-23.8 (1.48)
0.5	-23.2 (1.42)	-22.1 (3.14)
1	-23.1 (2.13)	-21.6 (1.33)
2	-23.0 (0.12)	-21.7 (-)
3	-22.1 (1.63)	-23.3 (-)

Supplementary table 4. Mean visual acuity in different NEI Vitreous Haze Grades

NEI Vitreous Haze Grade	Number of Eyes	Mean LogMAR vision (nearest value, SD)
**0**	**32**	**0.3 (0.5)**
**0.5**	**25**	**0.4 (0.6)**
**1**	**13**	**0.4 (0.7)**
**2**	**4**	**0.3 (0.3)**
**3**	**3**	**-0.5 (0.5)**
